# Phagocytosis of *Mycobacterium fortuitum* by Caprine Alveolar Macrophages Is Associated with iNOS and Pro-Inflammatory Markers Expression

**DOI:** 10.3390/ijms27031529

**Published:** 2026-02-04

**Authors:** Miriam Blay-Benach, Patricia Cuenca-Lara, Joan Repullés, Zoraida Cervera, Bernat Pérez de Val

**Affiliations:** 1 IRTA, Centre de Recerca en Sanitat Animal (CReSA, IRTA-UAB), Campus UAB, 08193 Bellaterra, Catalonia, Spain; patricia.cuenca@irta.cat (P.C.-L.); joan.repulles@irta.cat (J.R.); zoraida.cervera@irta.cat (Z.C.); 2 Unitat Mixta d’Investigació IRTA-UAB en Sanitat Animal, CReSA, Campus UAB, 08193 Bellaterra, Catalonia, Spain

**Keywords:** alveolar macrophages, phagocytosis, activation, innate immunity, *Mycobacterium fortuitum*, goat

## Abstract

Mycobacterial diseases, including tuberculosis (TB), remain the major health and economic challenges in livestock, underscoring the need to characterise the innate immune mechanisms involved in early bacterial containment. Alveolar macrophages (AMs) are the first line of defence against inhaled mycobacteria, yet the functional links between activation, polarisation, and phagocytic capacity in caprine AMs remain poorly defined. In this study, we compared a pH-dependent live-cell fluorescence assay with a culture-based method to evaluate phagocytosis and clearance of *Mycobacterium fortuitum* under different immunostimulatory conditions. AMs were stimulated in vitro with LPS or heat-inactivated *Mycobacterium bovis* (HIMB), and phagocytosis was assessed alongside activation and pro-inflammatory markers. Both approaches showed that LPS stimulation significantly enhanced mycobacterial clearance, despite reduced initial bacterial uptake. Moreover, this improved phagocytic capacity was associated with increased expression of the inducible Nitric Oxide Synthase (iNOS), MHCII, CD80, and CD86, as well as an elevated production of some pro-inflammatory cytokines. In contrast, HIMB induced cytokine secretion but failed to enhance activation markers or bacterial clearance. Collectively, these results establish the first association between pro-inflammatory activation and functional mycobacterial phagocytosis in caprine AMs and validate a robust methodological framework for studying innate immune responses relevant to TB and vaccine development in goats.

## 1. Introduction

The health and economic impact of mycobacterial diseases, such as tuberculosis (TB) or Johne’s disease in domestic ruminants [1,2,3,4,5], highlights the need to understand the immunological mechanisms underlying protection and vaccine development [6,7,8,9,10,11,12]. Macrophages play a central role in the early containment of mycobacterial infections. In TB, alveolar macrophages (AMs) are usually the first cells to encounter inhaled bacilli, and they largely determine whether the infection is controlled or progresses. Despite their relevance, studies on livestock remain scarce. This is especially true in goats, a highly susceptible species that can transmit mycobacteria to other livestock, wildlife, and humans [1,2,13].

AMs are key effector cells of the innate immune system that play a vital role in phagocytosis, a tightly regulated cellular mechanism responsible for the recognition, internalisation, and elimination of bacteria and apoptotic cells via the activation of different signalling pathways that allow changes in the acidic composition of the phagosome [14,15]. In the alveoli, AMs work together with other resident and infiltrating immune cells and undergo activation and polarisation to mediate their response [16,17]. Thus, the capacity of these cells to develop an efficient phagocytic cycle, coupled with their activation, is critical to limiting bacterial dissemination.

AMs are characterised by their plasticity and heterogeneity, which relies on their ability to polarise to either M1-classically activated AMs or M2-alternatively activated AMs [18]. The M1 pro-inflammatory profile is characterised by the expression of cytokines like TNFα, IL-6, IL-1β, as well as nitric oxide (NO) and other reactive oxygen species (ROS). This phenotype allows for a pathogen-killing environment during an active infection. In contrast, M2 AMs reveal an anti-inflammatory state that prevents tissue damage and performs apoptotic cell clearance [19,20,21]. The balance between the two phenotypes is essential for tissue homeostasis.

Notably, phagocytosis carried out by AMs should not be regarded as an isolated process but rather as a coordinated interaction of various mechanisms. Generally, confocal microscopy, flow cytometry, and spectrofluorometry are used as techniques for phagocytosis evaluation [22,23,24,25]. However, more sophisticated techniques for this aim are still needed to overcome limitations regarding the performance in high-throughput studies, as well as the assessment of phagocytosis at fixed time-points, due to the inability to monitor the process in real time. Recently, a culture-based assay was developed to evaluate mycobacterial elimination in cell lysates at different specific infection times [26]. Complementary to this, technologies like the Incucyte^®^ SX5 Live-Cell Imaging and Analysis System (Sartorius, Göttingen, Germany) enable monitoring of live phagocytosis based on labelled mycobacteria. Although fluorescently labelled *Escherichia coli* and *Staphylococcus aureus* particles are well established for phagocytosis assays [27], to our knowledge, the application of pH-dependent dyes to track mycobacterial uptake and acidification in real time has not been reported.

To address this gap, this study aimed to compare two complementary approaches to evaluate different stages of the phagocytosis of *Mycobacterium fortuitum*, a fast-growing, low-virulence non-tuberculous mycobacteria (NTM) [28,29,30] suitable for working under laboratory conditions, which has been used in vitro as a macrophage infection model in a few other studies [31,32].

This study evaluated how caprine AMs activation and pro-inflammatory states influence *M. fortuitum* phagocytosis by stimulating cells with heat-inactivated *Mycobacterium bovis* (HIMB), a known inducer of innate responses [33], as well as lipopolysaccharide (LPS), a widely used non-specific macrophage activator [34]. To our knowledge, this is the first study to address the relationship of pro-inflammatory activation with mycobacterial phagocytosis in goat AMs.

## 2. Results

### 2.1. Phagocytosis Performance of Caprine AMs

#### 2.1.1. LPS Stimulation of Caprine AMs Enhanced *M. fortuitum* Load Reduction

**Figure 1:**
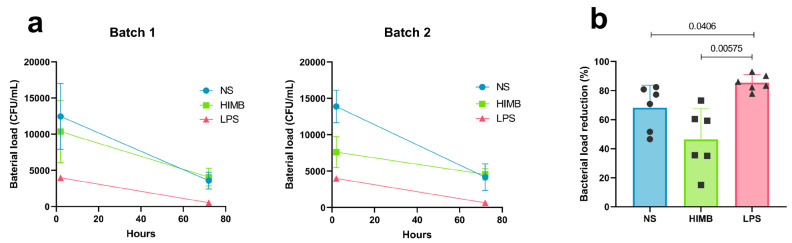
*M. fortuitum* phagocytosis detected using the cell-culture phagocytosis assay. (**a**) Bacterial load calculated with a standardised mathematical formula from cell lysates of differentially stimulated cells, cultured at 2 h and 72 h p.i. (**b**) Percentage of bacterial load reduction after 72 h of infection. Each dot represents a different macrophage batch replicate. Results represented as Mean ± SD. NS: non-stimulated, p.i.: post infection.

**Figure 2:**
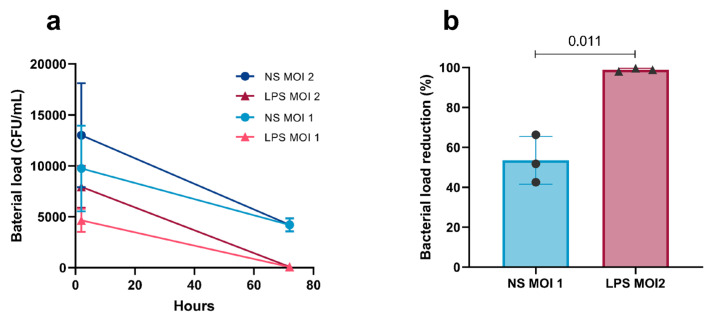
Cell internalisation correction of the culture-based phagocytosis assay. (**a**) Bacterial load calculated with a standardised mathematical formula from cell lysates of LPS and non-stimulated (NS) cells, infected with different MOIs of *M. fortuitum,* cultured at 2 h and 72 h p.i. (**b**) Percentage of bacterial load reduction, from NS cells infected at an MOI 1 and LPS-stimulated cells infected at an MOI 2 of *M. fortuitum*. Each dot represents a different replicate. Results represented as Mean ± SD. NS: non-stimulated, p.i.: post infection.

At 2 h post infection (p.i), non-stimulated (NS) cells had similar mycobacterial engulfment capacity in both batches (12,467.1 colony-forming units (CFU)/mL/13,874.8 CFU/mL). HIMB-stimulated cells showed variations in mycobacterial entry but slightly resembled the entry in NS cells (10,358.2 CFU/mL/7607.8 CFU/mL). In both batches, LPS-stimulated cells showed lower mycobacterial entrance compared to other stimuli (3982.7 CFU/mL, 3989.4 CFU/mL) (Figure 1a). To account for the differences in bacterial load entry, we calculated the percentage of bacterial load reduction after 72 h p.i. LPS-stimulated cells showed the highest mean reduction (85.43%) despite presenting a reduced initial engulfment, indicating a better capacity for eliminating mycobacteria. HIMB-stimulated cells, on the other hand, did not enhance bacterial clearance in goat AMs in either of the two batches (mean; 46.35%). Still, NS cells accomplished mycobacterial reduction with high efficiency (mean; 68.2%). LPS-stimulated cells showed statistically significant differences in the percentage of reduction compared to NS and HIMB-stimulated cells (*p*-values: 0.0406 and 0.00575) (Figure 1b). The second set of experiments aimed to normalise mycobacterial cell-internalisation by using batch one. Comparable levels of mycobacterial engulfment were achieved using an MOI 2 of *M. fortuitum* for LPS-stimulated cells (7942.47 CFU/mL) and an MOI 1 for NS cells (9752.06 CFU/mL), allowing for a precise comparison of phagocytic activity between the two groups (Figure 2a). LPS-stimulated cells exhibited statistically significant (*p*-value: 0.011, (98.8%)) greater mycobacterial clearance than NS cells (51.7%) as seen in Figure 2b. Thus, amplifying the differences in mycobacterial clearance after LPS stimulation observed in experiment one from 1.2 times to 1.9 times.

#### 2.1.2. pH-Dependent Labelling of *M. fortuitum* Enables Quantification of Live Phagocytosis in Stimulated AMs

**Figure 3:**
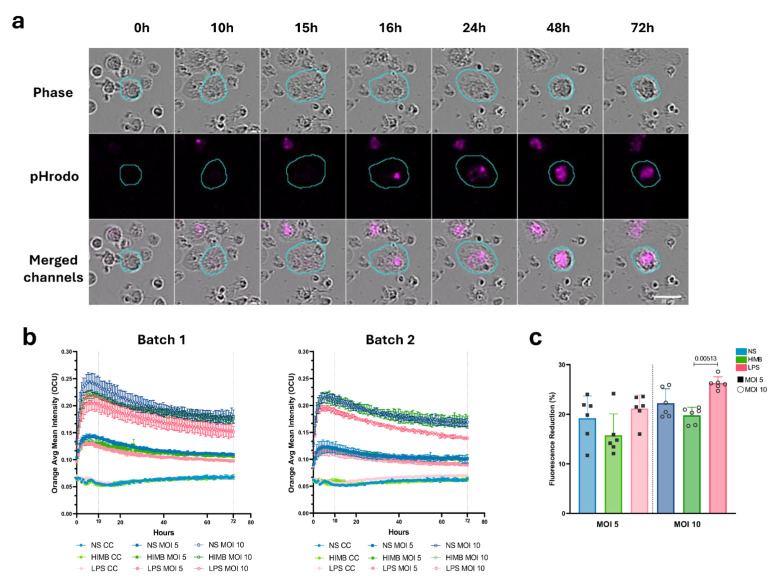
Phagocytosis assay using fluorescent live-cell analysis with Incucyte^®^ SX5. (**a**) An isolated alveolar macrophage monitored at different time-points (Full Video at Video S1). All channels show a cyan overline displaying the AI Cell Health segmentation mask. The internalised orange signal is represented in magenta. Scale bar at 25 µm. (**b**) Mean fluorescence intensity (OCU) of phagocytosing AMs, after the internalisation of labelled *M. fortuitum* in the phagolysosome, detected by the AI Cell Health Software module. Cells were infected either at an MOI 5 or 10 and were monitored for three days. (**c**) Percentage of fluorescent signal reduction from 10 h to 72 h post infection, of differentially stimulated AMs. Colour depth represents MOI increase. Results are represented as Mean ± SD. AMs: alveolar macrophages; NS: non-stimulated.

**Figure 4:**
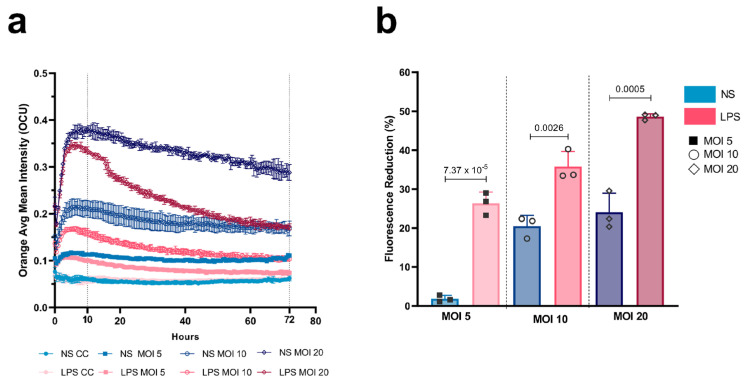
MOI increase in the Incucyte^®^ SX5 phagocytosis assay. (**a**) Mean fluorescent intensity (OCU) detection of phagocytosing AMs analysed by the AI Cell Health Software after infection with different MOIs of *M. fortuitum*. (**b**) Percentage of fluorescent signal reduction from 10 h to 72 h post infection. Colour depth represents MOI increase. Results are represented as Mean of replicates ± SD. AMs: alveolar macrophages; NS: non-stimulated.

Phagocytosis was evaluated by measuring the intracellular Orange Mean Fluorescence Intensity (OCU) in the segmented AMs as detected by the AI Cell Health software module on the Incucyte^®^ SX5 Live Cell System (Version 2022B Rev2). Images were acquired every hour for 3 days. Fluorescence levels in unlabelled cells (Control Cells (CCs)) remained low with values of OCU under 0.07. Figure 3a and Video S1 illustrate the temporal evolution of a phagocytosing AM over three days. Phagocytosis activity is indicated by the appearance of the orange signal (shown as magenta in the image) corresponding to the acidification of the pH-dependent label of *M. fortuitum*. Kinetics of mean fluorescence for each stimulus and MOI are shown in Figure 3b. Infecting macrophages at an MOI 5 and 10 showed a peak of intensity at 10 h p.i. followed by a mild decrease until the end of the experiment. Differences between stimuli were compared by the percentage of fluorescence reduction between 10 and 72 h (Figure 3c). LPS-stimulated cells exhibited the highest percentage of signal reduction independently of the MOI—21.14% for MOI 5 and 26.4% for MOI 10—with MOI 10 being statistically different from HIMB-stimulated cells (*p*-value 0.00513). Following this, NS cells presented reductions of 19.1% and 22.2%, respectively. HIMB-stimulated cells showed the lowest percentage of signal reduction (15.7% and 19.7%). An MOI increase to 20 was performed to amplify the OCU signal, allowing a higher differentiation of the slope in the LPS curves (Figure 4a, Table 1). Moreover, statistical differences (*p*-values MOIs 5 to 20; 7.37 × 10^−5^, 0.0026, 0.0005) between LPS-stimulated cells and NS cells in the fluorescence reduction were present for all MOIs, suggesting that LPS-stimulated cells possessed a consistently enhanced ability to phagocytose *M. fortuitum* (Figure 4b). AMs monitoring and segmentation over time are shown in Video S2.

### 2.2. AMs In Vitro Stimulation with HIMB and M. fortuitum Led to an Increased Pro-Inflammatory Cytokine Profile

**Figure 5:**
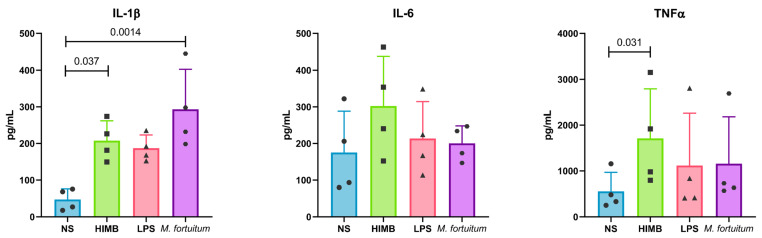
Luminex multicytokine assay. Production of three pro-inflammatory cytokines by AMs after 24 h of in vitro stimulation. Each dot represents an independent macrophage batch. Results are represented as Mean ± SD. AMs: alveolar macrophages; NS: non-stimulated.

After 24 h of stimulation, cytokine levels were consistently higher in stimulated cells compared to non-stimulated ones across all cytokines analysed (Figure 5). IL-1β expression was significantly more elevated (*p*-value: 0.0014) in *M. fortuitum*-infected cells compared to non-stimulated ones. HIMB-stimulated cells showed significantly (*p*-value: 0.037) increased levels of IL-1β as well. Additionally, LPS stimulation elevated IL1-β production, although not statistically significantly when compared to non-stimulated AMs. HIMB-stimulated cells exhibited slightly higher production of IL-6 and TNFα compared to other stimuli but only reached statistical significance for TNFα production (*p*-value: 0.031).

### 2.3. The Inducible Nitric Oxide Synthase (iNOS) and MHCII Are Good Indicators of Improved Phagocytic Capacity in Stimulated AMs

**Figure 6:**
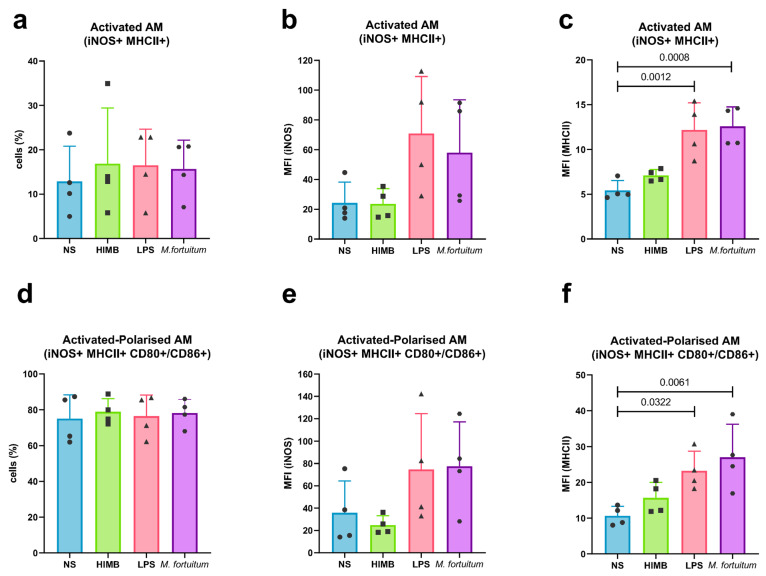
Flow cytometry analysis of activation and polarisation profiles of stimulated Ams. (**a**) Percentage of activated AMs (iNOS+ MHCII+). (**b**) MFI of intracellular iNOS in activated cells. (**c**) MFI of surface MHCII in activated cells. (**d**) Percentage of activated-polarised AMs (iNOS+ MHCII+ CD80+/CD86+). (**e**) MFI of iNOS in active-polarised AMs. (**f**) MFI of MHCII in active-polarised AMs. Each dot presents an independent macrophage batch. Results are represented as Mean ± SD. AMs: alveolar macrophages; MFI: mean fluorescent intensity; NS: non-stimulated.

Flow cytometry on adherent cells was performed to assess their activation and polarisation states following stimulation by detecting both surface and intracellular markers. Cell activation was defined by the co-expression of the iNOS and MHCII. Twenty-four hours post stimulation, all groups exhibited similar percentages of activated cells (Figure 6a). Analysis of the mean fluorescence intensity (MFI) of each marker separately in activated cells revealed that LPS and *M. fortuitum*-stimulated AMs showed increased iNOS (Figure 6b) and MHCII expression (Figure 6c). However, statistically significant differences were observed only for MHCII expression in LPS and *M. fortuitum* groups when compared with NS cells (*p*-values: 0.0012 and 0.0008, respectively). Accounting for the subpopulation of polarised cells (CD80+ and/or CD86+) within active cells yielded comparable results. Here, all stimulants presented similar active-polarised profiles equivalent to those in the NS cells (Figure 6d). Nevertheless, assessment of the relative expression of iNOS indicated the greatest increases in the LPS and *M. fortuitum* groups, despite presenting no statistical significance (Figure 6e). MHCII expression within this subpopulation showed statistical differences between LPS (*p*-value: 0.0322) and *M. fortuitum* (*p*-value: 0.0061) groups when compared to NS cells (Figure 6f). Contrarily, HIMB stimulation did not raise the MFI levels in either of the markers.

## 3. Discussion

AMs capacity to effectively phagocytose mycobacteria prior to infection is established as a key component of host defence against TB, given that these cells are the primary cellular target of the pathogen [35]. However, there is still a significant need to discern whether immunostimulants are effective in inducing functional changes able to modulate phagocytosis in caprine AMs. Integrating phagocytic performance, activation and polarisation states, and the capacity of AMs to develop trained immunity offers a robust framework for evaluating how different immunostimulatory compounds modulate immune and functional responses in the lung mucosa.

Using two complementary phagocytosis assays, this study showed that stimulus-enhanced phagocytic capacity, encompassing phagosome acidification and bacterial lysis, was associated with iNOS production and pro-inflammatory activation profile in caprine AMs.

The results demonstrated that LPS significantly enhanced mycobacterial clearance when compared to control cells and HIMB-stimulated cells, as detected by both techniques, particularly when accounting for the reduced bacterial uptake initially observed. Both approaches helped us distinguish between the lower mycobacterial entry into the cell in LPS-stimulated AMs and their enhanced capacity to reduce *M. fortuitum* CFU equivalents at 72 h or intracellular fluorescence of pHrodo^®^-labelled *M. fortuitum* over time, suggesting bacterial clearance. In addition, LPS stimulation of AMs induced a mild increase in IL-1β and TNFα production compared with non-stimulated AMs, although this difference did not reach statistical significance. This non-significant trend may reflect the use of a single time-point measurement, as the expression of these cytokines is known to peak at earlier stages following stimulation [36]. Despite this limitation, the observed cytokine trends are consistent with prior findings in porcine alveolar macrophages [37] and murine peritoneal macrophages, where LPS stimulation similarly induced the production of pro-inflammatory cytokines [38]. Comparable responses have also been documented in human macrophage cell lines, where phagocytosis of IgG-coated beads in LPS-stimulated cells leads to an increase in TNFα production [39]. Generally, LPS stimulation has been associated with an M1 pro-inflammatory profile [40,41], although in certain conditions it has also been described to promote the induction of M2b macrophages [42]. Interestingly, while LPS primarily induces pro-inflammatory activity, phagocytosis using human and murine macrophages has traditionally been related to M2 macrophages, particularly due to their role in clearing cell debris and apoptotic cells as part of the anti-inflammatory response aimed at maintaining tissue homeostasis [21,27,43,44]. However, M1 polarised AMs have been shown to have enhanced capacity to phagocytose nanoparticles when compared to M2 macrophages, despite knowing some mechanisms can evade M1-macrophage-mediated phagocytosis [45]. M1 activity leads to increased acidification in the phagosome, improving the cell bactericidal activity in infections such as those of *M. tuberculosis* [46]. These results align perfectly with our correlation of phagocytosis with an M1 pro-inflammatory state, underscoring the functional plasticity of macrophages and suggesting that the phagocytic activity is not strictly confined to a single polarisation state.

Furthermore, LPS-stimulated AMs induced higher expression of iNOS and MHCII, both related to macrophage activation. These results revealed a strong association between upregulation of these functional biomarkers and the expression of M1-associated surface markers, particularly in LPS-stimulated AMs. iNOS plays a key role in macrophage bactericidal function by generating NO, which has a potent antimycobacterial capacity through the production of different reactive oxygen and nitrogen species [47]. Analysing iNOS together with MHCII, which is essential for antigen presentation to T-helper cells, provides a meaningful indicator of enhanced predisposition of AMs as effector cells for bacterial elimination. Notably, the activation profile (determined by both iNOS and MHCII expression) of LPS-stimulated cells closely resembled that of *M. fortuitum*-infected macrophages, which served as a reference for actively phagocytosing cells. These results, aligned with the observed increase in the phagocytic capacity of LPS-stimulated AMs, reinforce the relevance of iNOS and MHCII as markers of improved activation and their possible association with phagocytosis. Similar results were obtained when analysing the subpopulation of CD80+ and/or CD86+ cells within activated AMs. These results correlate with the reduced dissemination of *M. tuberculosis* to different cell types observed in mice with activated AMs following immunisation with the intranasal Bacillus Calmette-Guerin (BCG) vaccine [48].

In contrast, HIMB stimulation was not associated with enhanced expression of the activation or M1-polarisation markers assessed, nor with improved bacterial clearance in any of the assays performed, despite inducing a significant increase in the production of pro-inflammatory cytokines, especially IL-6 and TNFα. This observation suggests that pro-inflammatory cytokine secretion alone is not sufficient to promote effective mycobacterial elimination, as neither of the two macrophage batches demonstrated improved phagocytosis following HIMB stimulation. In accordance with this, following HIMB stimulation, the expression of iNOS and MHCII within activated cell subsets remained at levels comparable to those observed in non-stimulated cells. Differences in the activation induced by HIMB or *M. fortuitum* likely reflect the structural diversity in the composition of the lipoglycan bacterial cell wall. Pathogenic MTBC species express mannose-capped lipoarabinomannan (ManLAM) linked to the inhibition of pro-inflammatory pathways and phagosome maturation, whereas fast-growing mycobacteria express phosphor-myo-inositol-capped LAM (PILAM), which elicits stronger pro-inflammatory signals in AMs [49,50]. These structural variations provide a possible molecular explanation of the cytokine profiles and activation phenotypes observed in caprine AMs. The rationale for using mycobacteria-related stimuli such as HIMB is based on its demonstrated immunostimulatory activity in ex vivo assays [51], which led us to hypothesise that it could also enhance phagocytic responses. HIMB has also been shown to induce protective immune responses in different in vivo animal models, including mice [52], pigs [53], and also goats [8]. However, the results of this study indicated that the immunostimulant capacity of HIMB is not necessarily associated with improved phagocytosis in vitro. Likewise, some vaccines against TB, including HIMB or the live-attenuated *M. bovis* BCG, have demonstrated the ability to induce epigenetic changes in innate immune cells, conferring broad-spectrum non-specific protection against other pathogens that resemble the memory-like effect of adaptive immune cells. This concept has been coined as trained immunity, and it is crucial to understand the cross-protective effects of some immunogens leading to a stronger innate immune response [7,48,53,54].

Remarkably, the comparative analysis of a real-time imaging technique based on pH-dependent labelling of mycobacteria with a culture-based assay employing the BACTEC™ MGIT™ system yielded consistent outcomes and provided a comprehensive assessment of phagocytosis in caprine AMs. The labelled mycobacteria imaging analysis showed higher throughput, greater flexibility in data interpretation, and simpler workflow, making it more suitable for large-scale studies. By employing both methodologies, we established that the percentage of reduction in bacterial load and fluorescence intensity served as reliable indicators of phagocytic activity in goat AMs. A very similar culture-based phagocytosis assay was previously used to evaluate the mycobacterial phagocytosis efficiency of bovine macrophages derived from monocytes isolated from bovine blood [26].

Employment of *M. fortuitum* as the infection strain demonstrated robust and reproducible results. Although *M. fortuitum* does not reproduce the pathological features of caprine TB, its shared structural characteristics with other mycobacteria make it suitable as a preliminary model for establishing and optimising the interactions between AMs and mycobacteria. In this report, we present data obtained using high MOIs of *M. fortuitum* for Incucyte^®^-based infection assays. MOIs were initially calculated from the theoretical frozen stock titres. However, based on our measurements, the efficiency of the fluorescent labelling was approximately 25%. Lastly, the 2–3 h doubling time for this NTM [55] is sufficiently slow to maintain a stable fluorescent signal and cell culture condition throughout the monitored phagocytosis window, preventing artefacts from rapid bacterial replication.

Through this study, the use of the Incucyte^®^ SX5 Live-Cell System was successfully validated for *M. fortuitum* phagocytosis assessment in caprine AMs. Furthermore, this study addresses a relevant gap in the field of immune responses against pulmonary infectious diseases, such as TB, where relationships between the phagocytic capacity and AMs phenotypic signatures remain poorly characterised, particularly in livestock species. Given that goats represent a suitable translational animal model for assessing new TB vaccine candidates [56,57,58], a deeper understanding of vaccine-induced innate immune mechanisms is essential for the development of more effective vaccines aimed at enhancing innate immunity in the lung mucosa.

## 4. Materials and Methods

### 4.1. Reagents

AMs were cultured in complete RPMI medium (cRPMI), which consisted of RPMI-1640 containing 10% SBF, 1% Glutamine, 1% Streptomicin/Penicillin, 0.5% Nystatin. Lipopolysaccharides from *Escherichia coli*, LPS, serotype O55:B5 (Sigma Aldrich, St. Louis, MO, USA). Phosphate-Buffer Saline (PBS) and Hanks Balanced Salt Solution (HBSS) (Corning, Corning, NY, USA). Triton X-100 (Sigma Aldrich).

### 4.2. Bacterial Strains

*Mycobacterium fortuitum* (ATCC 6841) was used for AMs infection. Preserved in BHI + 20% glycerol and stored at −80 °C. HIMB was produced at NEIKER (Derio, Spain) as previously described by Garrido et al., 2011 [33].

### 4.3. Ethical Declarations

Animals were purchased under TB-free conditions at the Servei de Granges i Camps Experimentals (SGCE) of the Autonomous University of Barcelona with registration number B9900042. The experimental procedures were approved on 19 June 2024 by the Animal Welfare Committee of the Autonomous University of Barcelona (Procedure No. 5482-CEEAH-UAB) and the Generalitat de Catalunya (Reference No. 12164), in accordance with European Union regulations on the protection of experimental animals. This study was carried out in full compliance with the ARRIVE guidelines.

### 4.4. Bronchoalveolar Lavage (BAL) for AMs Isolation

BAL was performed to isolate AMs from the lungs of healthy non-vaccinated young goats. Animals were humanely euthanised by intravenous administration of pentobarbital at 200 mg/kg. After euthanasia, the trachea was tied, and the lungs were carefully removed. Subsequently, lungs were gently lavaged with 500 mL of sterile PBS + 0.1% gentamicin by a light massage to avoid epithelial cell contamination. BAL was centrifuged at 380× *g* for 15 min at 4 °C. Pellets were then washed three times with sterile PBS and pooled. Hypotonic lysis was performed to remove red blood cells. Cells were counted using Trypan Blue and cultured in a T25 flask to assess contamination and morphology after 24 h. The remaining cells were cryopreserved using CryoStor^®^ (STEMCELL Technologies, Vancouver, BC, Canada) and stored in liquid nitrogen for further use.

### 4.5. Ex Vivo Stimulation of Caprine AMs

AMs were thawed in the bath at 37 °C for two minutes. Next, cells were centrifuged and resuspended in the desired amount of cRPMI and counted to assess cell viability using Trypan Blue. Cells were left to adhere for two hours in 200 µL of media in a 96-well flat-bottom plate or in 1 mL in 24-well plates.

After two hours of incubation, the cell media was removed and AMs were stimulated as follows: for phagocytosis, cells were incubated with culture medium only as negative controls (NS), stimulated with 100 ng/mL LPS and infected at a 10:1 multiplicity of infection (MOI) with HIMB for 24 h at 37 °C and 5% CO_2_. In addition, cells were infected at MOI 5 with *M. fortuitum*, for the detection of activation and pro-inflammatory markers for 24 h at 37 °C and 5% CO_2_. After incubation, supernatants were collected and stored at −20 °C.

### 4.6. Experimental Design

Two sets of experiments were performed using caprine AMs stimulated in vitro for phagocytosis analysis.

#### 4.6.1. Experiment One

For this experiment, macrophages from two different goats were used, and cells were stimulated for 24 h with LPS and HIMB as described above or maintained with cell culture media only as non-stimulated controls. For the culture-based assay, cells were infected with *M. fortuitum* at an MOI 1 for all conditions. For the Incucyte^®^ fluorescence-dependent assay, cells were infected with either MOI 5 or 10 and maintained uninfected with cRPMI as cell controls (CCs).

#### 4.6.2. Experiment Two

One of the two goats was selected for this experiment. Cells for all phagocytosis assays were stimulated with LPS as defined above and maintained unstimulated with cRPMI only. In vitro infection for the culture-based assay was performed at MOIs 1 or 2. In addition, infection was performed at MOIs 5, 10, or 20 for the Incucyte^®^ assay. Non-infected controls were added for all experiments.

### 4.7. Phagocytosis Assays

#### 4.7.1. Culture-Based Phagocytosis Assay

This protocol was adapted from Juste et al., 2016 [26]. A total of 1 × 10^5^ cells/well were added to a 96-well plate and stimulated and infected as described above. After 2 h, cells designated for the 72 h p.i. assay were washed with cRPMI and incubated again at 37 °C with 5% CO_2_. Supernatants from cells at 2 h p.i. (time 0) were removed, and cells were washed twice with 200 µL HBSS. Cell lysis was performed by adding 200 µL of 0.1% Triton X-100 for 10 min at RT and firmly pipetting to retrieve the lysed suspensions. Cell lysates were centrifuged at 20,000× *g* for 10 min and resuspended in 1 mL PBS. Mycobacteria Growth Indicator Tubes (MGITs) were supplemented with BACTEC™ MGIT™ 960 Supplement Kit (BD, Franklin Lakes, NJ, USA) and inoculated with 100 µL of the bacterial suspensions. The procedure was repeated after three days for the 72 h p.i. cells. Lysates were incubated in the BACTEC™ MGIT™ instrument until the time of detection (TTD) of the tube was obtained. The equivalent bacterial load detected was calculated using a robust mathematical formula which correlates TTD (in minutes) to the log of CFUs made with a serial dilution of the same *M. fortuitum* strain (Figures S1 and S2). The percentage of bacterial reduction was calculated by the division of the difference in CFUs at time 2 h p.i. and that at 72 h p.i. divided by the CFU count at time 2 h p.i. This protocol focused on mycobacterial internalisation and lysis by quantifying the recovered bacterial loads.

#### 4.7.2. Fluorescence Microscopy-Based Phagocytosis Assay

To validate this assay, we successfully labelled a strain of *M. fortuitum* with orange pHrodo^®^ (Sartorius, Göttingen, Germany), a pH-dependent dye that allowed for the detection of fluorescent signal upon the acidification and maturation of the phagolysosome [59]. For *M. fortuitum* labelling, the pHrodo^®^ Orange Cell Labeling Kit for Incucyte^®^ was used following the manufacturer’s protocol with minor adjustments. A mycobacterial suspension was centrifuged at 2460× *g* for 30 min and resuspended in 5 mL of Wash Buffer. The cells were then centrifuged under the same conditions and resuspended in pHrodo^®^ Labeling Buffer to a cell density of 1 to 2 × 10^6^ cells/mL approximately. Next, the bacterial suspension was labelled with 250 ng/mL of pHrodo^®^ Cell Labeling Dye for Incucyte^®^ for 1 h at 37 °C. Before infection, the labelled mycobacterial suspension was centrifuged and washed with cell culture media.

For the phagocytosis assay, 5 × 10^4^ cells/well were plated in a 96-well plate and stimulated as described. On day two, cells were washed with 200 µL of PBS before infection with the labelled mycobacteria right before entering the Incucyte^®^ SX5Live-Cell Analysis System. Non-infected controls were included for all conditions to account for background noise. Four images per well from three replicates per condition were captured every hour for three days using the 20× objective. Acquisition time for the orange channel was 700 ns. For correct cell recognition, segmentation sensibility was established at 0.7, and the orange channel was segmented using Top-Hat with no mask configuration with a radius equal to 25 µm. Furthermore, only macrophages with areas greater than 80 µm^2^ were considered for inclusion in the analysis.

To evaluate phagocytosis, the curves for the OCU were monitored for 72 h and analysed using the AI Cell Health integrated software module using the phase and orange channels. The 10 h time-point was set as the threshold for calculating the percentage of fluorescence reduction since, at this stage, most cells had already reached the fluorescence peak and entered the decline phase to be analysed.

### 4.8. Multicytokine Measurement

In total, 10^6^ cells/well in 24-well plates from four different non-vaccinated animals were cultured and stimulated for 24 h with 100 ng/mL of LPS, MOI 10 with HIMB, or MOI 5 with *M. fortuitum*. Supernatants were removed and stored until the analysis. Cytokines were analysed using a custom-made bovine multiplex assay kit (MILLIPLEX^®^ Millipore, Merck Life Science, Darmstadt, Germany) for pro-inflammatory cytokines (IL-6, IL1β, TNFα) following the manufacturer’s instructions. Plates were read using MAGPIX system with xPONENT software version 4.3 (Thermofisher Scientific, Waltham, MA, USA) software for results visualisation.

### 4.9. Expression of Activation and Polarisation Markers for Functional Flow Cytometry Analysis

AMs from four non-vaccinated animals were thawed and stimulated in non-treated 96-well plates as previously described. After 6 h of incubation, Brefeldin A was added to a final concentration of 10 µg/mL. On day two, supernatants were removed, and cells were detached from plates by adding 200 µL of PBS for 20 min at RT. Detached cells were carefully transferred to a 96-well round-bottom plate. Following this, AMs were stained as listed in Table 2. For intracellular iNOS staining, AMs were permeabilised with Leucoperm. Lastly, stained cells were fixed with 1% paraformaldehyde and read using the MACSQuantify^TM^ instrument (Miltenyi Biotec, Bergisch Gladbach, Germany) for flow cytometry analysis. The gating strategy is shown in Figure S3.

### 4.10. Statistics and Data Analysis

Analysis was performed to compare responses in stimulated groups against non-stimulated samples. All results are expressed as mean ± standard deviation (SD). Statistical analysis was performed using GraphPad Prism 8.3.0 software.

Normality, for all tests performed, was analysed using Shapiro–Wilk test. Pairwise comparisons (LPS-stimulated cells vs. NS cells) used unpaired one-tailed *t*-tests (Welsch’s correction for bacterial load reduction). For multiple group comparisons: percentage of bacterial load reduction data was analysed using an unpaired *t*-test with Welch’s correction, without correction for multiple comparisons. Fluorescence reduction and pro-inflammatory cytokine data were analysed using a non-parametric Kruskal–Wallis test followed by Dunns’s post hoc without correction for multiple comparisons. Flow cytometry analysis of activation and polarisation markers was performed using one-way analysis of variance (ANOVA) with Bonferroni’s multiple comparison post hoc test. Statistical significance was considered if the *p*-value < 0.05.

## Figures and Tables

**Table 1:** Slope values of mean fluorescence intensity kinetics in the Incucyte^®^ SX5 phagocytosis assay calculated from 10 to 72 h p.i.

SLOPE (10 h–72 h)	MOI 5		MOI 10		MOI 20	
	NS	LPS	NS	LPS	NS	LPS
Batch 1	−0.0001150	−0.0003554	−0.0006190	−0.0008024	−0.001395	−0.002445

**Table 2:** Reagents for the functional markers analysis by flow cytometry.

Marker	Fluorochrome	Isotype	Clone	Source	Notes
*Viability marker*	Viobility 405/520	-	Fixable Dye	Miltenyi Biotec	
*Bovine CD80*	FITC	Mouse, IgG1	IL-A159	ThermoFisher Scientific	
*Bovine CD86*	PE	Mouse, IgG1	IL-A190	ThermoFisher Scientific	
*Mouse iNOS*	Alexa Fluor 647	Mouse, IgG1	4E5	ThermoFisher Scientific	Manually Conjugated
*B* *ovine MHCII*	NovaFluor 755	Mouse, IgG2a	IL-A21	ThermoFisher Scientific	Manually Conjugated

## Data Availability

The original contributions presented in this study are included in the article/Supplementary Materials. Further inquiries can be directed to the corresponding authors.

## Supplementary Materials

The following supporting information can be downloaded at https://www.mdpi.com/article/10.3390/ijms27031529/s1.

## Abbreviations

The following abbreviations are used in this manuscript:

AMs	Alveolar Macrophages
CC	Control Cells
CFU	Colony-Forming Units
HIMB	Heat-inactivated *Mycobacterium bovis*
LPS	Lipopolysaccharide
MFI	Mean Fluorescence Intensity
MGIT	Mycobacteria Growth Indicator Tubes
MOI	Multiplicity of Infection
NO	Nitric Oxide
iNOS	Inducible Nitric Oxide Synthase
ROS	Oxygen Reactive Species
OCU	Orange Mean Intensity Object Average
p.i.	Post infection
TB	Tuberculosis
