# Supplementary material for: Phagocytosis of Mycobacterium fortuitum by Caprine Alveolar Macrophages Is Associated with iNOS and Pro-Inflammatory Markers Expression
Source: Int J Mol Sci. 2026 Feb 4;27(3):1529. doi: 10.3390/ijms27031529 (PMC12897857; doi:10.3390/ijms27031529)
Supplement: Supplementary file 1 [file ijms-27-01529-s001.zip › Supplementary Figures.pdf]

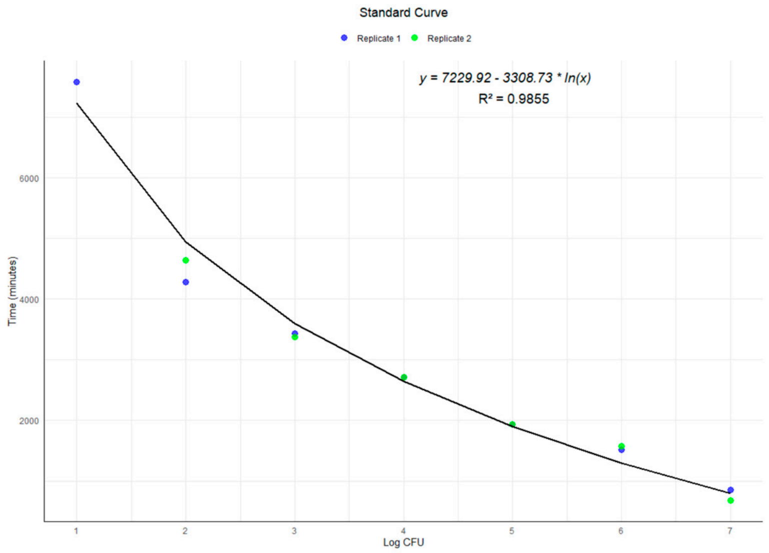

Figure S1. Standard curve of the culture-based assay, Experiment 1.

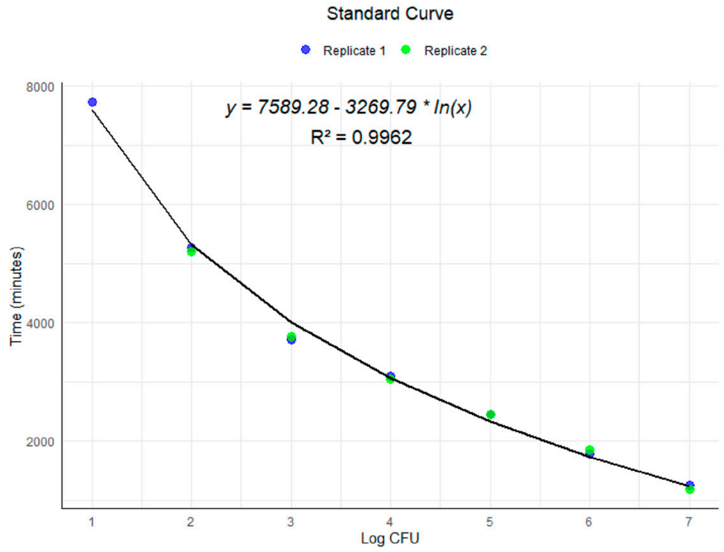

Figure S2. Standard curve for the culture-based assay, Experiment 2.

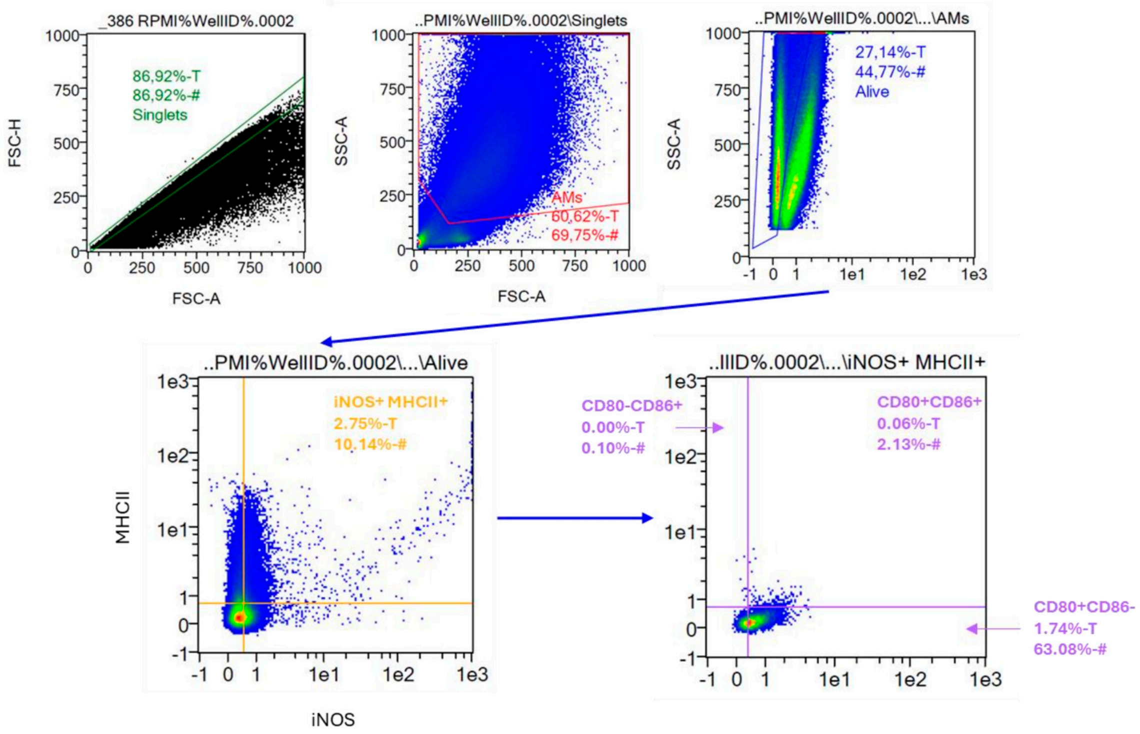

Figure S3. Gating Strategy for Activation-Polarisation FCA panel.

Video S1. Alveolar Macrophage monitoring over a three-day period.

Video S2. AMs segmentation and monitoring over a three-day period.
